# Whole-Genome Sequencing of *Sphingobium* sp. Strain RSMS, a Highly Efficient Tributyl Phosphate-Degrading Bacterium

**DOI:** 10.1128/MRA.00600-20

**Published:** 2020-10-15

**Authors:** Shyam Sunder Rangu, Ashish Beck, Mohak Sharda, Rita Mukhopadhyaya, Aswin Sai Narain Seshasayee, Devashish Rath

**Affiliations:** a Molecular Biology Division, Bhabha Atomic Research Centre, Mumbai, India; b UM-DAE Centre for Excellence in Basic Sciences, Mumbai, India; c National Centre for Biological Sciences, Tata Institute of Fundamental Research, Bangalore, India; d School of Life Science, The University of Trans-Disciplinary Health Sciences and Technology, Bengaluru, Karnataka, India; e Homi Bhabha National Institute, Anushaktinagar, Mumbai, India; University of Maryland School of Medicine

## Abstract

*Sphingobium* sp. strain RSMS was described earlier as an efficient degrader of tributyl phosphate, an organic pollutant. This report describes the generation and annotation of the genome sequence of *Sphingobium* sp. strain RSMS, which will facilitate future studies to identify genetic elements responsible for the degradation of tributyl phosphate.

## ANNOUNCEMENT

Widespread use of large volumes (∼3,000 to 5,000 tons/year) of tributyl phosphate (TBP) in the nuclear industry for extraction of uranium and plutonium (1) and in other industries (2) has resulted in large volumes of TBP-containing waste, which is an environmental hazard. Various bacterial species have been studied for TBP degradation (3–8). Among these, *Sphingobium* sp. strain RSMS has turned out to be most efficient in degrading TBP (7). To date, the genetic basis of TBP biodegradation has not been elucidated.

In this report, we present the complete genome sequence of RSMS, which was originally isolated from a TBP storage site in Bhabha Atomic Research Centre (Mumbai, India) (7). RSMS was inoculated into a modified mineral medium (7) supplemented with 1% glucose and 5 mM KH_2_PO_4_ (as the sources of carbon and phosphorus, respectively) and grown aerobically at 30°C. The culture grown overnight was harvested, and the genomic DNA was isolated using the GenElute bacterial genomic DNA kit (Sigma).

The whole-genome library was constructed using the Illumina TruSeq Nano DNA LT sample preparation kit set B according to the manufacturer’s instructions. A total of 11,610,225 paired-end 100-bp reads were generated from an Illumina HiSeq 2500 system, and low-quality reads were filtered using Trimmomatic v0.39 (9) with custom filters (Q score, >15 in a sliding window of 4 nucleotides [nt]; minimum length, >36 nt). For long reads, Oxford Nanopore Technologies 1D native barcoding genomic DNA kits (SQK-LSK108 and EXP-NBD103) were used to prepare the library according to the manufacturer’s instructions but without a DNA fragmentation step. A total of 80,396 long reads were obtained with a MinION flow cell (MIN106D). Live base calling was performed using MinKNOW v1.2.8 (Oxford Nanopore Technologies), and Albacore v2.0.2 (Oxford Nanopore Technologies) was used for demultiplexing. Canu v1.7 (10) was used for error correction. A hybrid *de novo* assembly was performed with long-read and short-read data in the Galaxy server (https://usegalaxy.org) using the Unicycler hybrid assembler (11) with default parameters. After assembly, the GC content was determined with QUAST v4.4 (12). Results similar to those described below were also obtained when the Nanopore reads were assembled using Canu v1.7 with default parameters. Sequence errors were corrected by mapping Illumina reads to this assembly.

The assembly of combined Nanopore (30× coverage) and Illumina HiSeq (226× coverage) reads yielded six replicons, i.e., chromosome 1 (3,744,159 bp, 3,561 coding sequences [CDSs]), chromosome 2 (1,152,427 bp, 1,001 CDSs), and four plasmids, namely, pRSM1 (152,994 bp, 140 CDSs), pRSM2 (34,461 bp, 37 CDSs), pRSM3 (26,288 bp, 26 CDSs), and pRSM4 (17,430 bp, 11 CDSs). Except for pRSM4, all replicons were obtained as single circular contigs. The chromosomes were rotated to start at the *dnaA* alleles. A complete genome size of 5.12 Mb and a GC content of 64.6% were obtained. Gene annotation using Prokka v1.44.0 (13) with default parameters showed 4,776 CDSs, 9 rRNA genes, and 62 tRNA genes.

### Data availability.

The GenBank accession numbers for the genome sequence of *Sphingobium* sp. strain RSMS are CP053222, CP053223, CP053224, CP053225, CP053226, and CP053227. The whole-genome sequencing raw records have been deposited in the SRA under BioProject accession number PRJNA505139.
